# Electrophysiological classification of *CACNA1G* gene variants associated with neurodevelopmental and neurological disorders

**DOI:** 10.3389/fphar.2025.1613072

**Published:** 2025-10-02

**Authors:** Amaël Davakan, Leos Cmarko, Barbara Ribeiro Oliveira-Mendes, Claire Bernat, Najlae Boulali, Jérôme Montnach, Stephanie E. Vallee, Mary B. Dinulos, Lydie Burglen, Vincent Cantagrel, Norbert Weiss, Sophie Nicole, Arnaud Monteil, Michel De Waard, Philippe Lory

**Affiliations:** ^1^ IGF, Université de Montpellier, CNRS, INSERM, Montpellier, France; ^2^ LabEx ‘Ion Channel Science and Therapeutics’, Montpellier, France; ^3^ Nantes Université, CNRS, INSERM, l’Institut du Thorax, Nantes, France; ^4^ Department of Pathophysiology, Third Faculty of Medicine, Charles University, Prague, Czechia; ^5^ Institute of Biology and Medical Genetics, First Faculty of Medicine, Charles University, Prague, Czechia; ^6^ Department of Pediatrics and Clinical Genetics, Dartmouth-Hitchcock Medical Center and Clinics, Lebanon, NH, United States; ^7^ Developmental Brain Disorders, Laboratory, Imagine Institute, Université Paris Cité, INSERM UMR1163, Paris, France; ^8^ Pediatric Neurogenetics Laboratory, Department of Genetics, Armand-Trousseau Hospital, APHP, Sorbonne University, Paris, France

**Keywords:** calcium channel, SCA42ND, electrophysiology, automated patch-clamp, Cav3.1

## Abstract

This study highlights the complementarity of automated patch-clamp (APC) and manual patch-clamp (MPC) approaches to describe the electrophysiological properties of eighteen Ca_v_3.1 calcium channel variants associated with various neurological conditions. Current density was measured efficiently for all variants in APC experiments, with four variants (p.V184G, p.N1200S, p.S1263A and p.D2242N) showing elevated current densities, compared to wild-type Ca_v_3.1 channel, while six variants (p.M197R, p.V392M, p.F956del, p.I962N, p.I1412T, and p.G1534D) displayed reduced current densities, and were therefore preferentially studied using MPC. The electrophysiological properties were well preserved in APC (e.g., inactivation and deactivation kinetics, steady-state properties), with only the APC-MPC correlation for activation kinetics being less robust. In addition, neuronal modeling, using a deep cerebellar neuron (DCN) environment, revealed that most of the variants localized to the intracellular gate (S5 and S6 segments) could increase DCN spike frequencies. This DCN firing was highly dependent on current density and further pointed to the gain-of-function (GOF) properties of p.A961T and p.M1531V, the two recurrent variants associated with Spinocerebellar Ataxia type-42 with Neurodevelopmental Deficit (SCA42ND). Action-potential (AP) clamp experiments performed using cerebellar and thalamic neuron activities further established the GOF properties of p.A961T and p.M1531V variants. Overall, this study demonstrates that APC is well-suited for high-throughput analysis of Ca_v_3.1 channel variants, and that MPC complements APC for characterizing low-expression variants. Furthermore, *in silico* modeling and AP clamp experiments reveal that the gain- or loss-of-function properties of the variants are determined by how the Ca_v_3.1 channel decodes the electrophysiological context of a neuron.

## Introduction

The T-type calcium channels (Ca_v_3.1, Ca_v_3.2 and Ca_v_3.3) are voltage-gated calcium channels with some unique features, notably their activation by low plasma membrane depolarization (low-voltage activated) and exhibiting fast inactivation (Zamponi et al., 2015; Lory et al., 2020). The Ca_v_3.1 channel is expressed in many types of neurons, from the cerebellum, thalamus and cortex, and contributes to neuronal excitability, especially rebound burst firing (Perez-Reyes, 2003). Several variants in *CACNA1G*, the gene encoding Ca_v_3.1 channels, are associated with neurological conditions, mainly cerebellar and neurodevelopmental disorders, including SCA42 (spinocerebellar ataxia type 42; (Coutelier et al., 2015)), SCA42ND (SCA42 with neurodevelopmental deficits; (Chemin et al., 2018)), and developmental and epileptic encephalopathy (DEE; (Berecki et al., 2020)). Aside from SCA42, most of these recently reported Ca_v_3.1 variants are *de novo* missense variants that greatly alter the biophysical properties of the Ca_v_3.1 channel (Lory et al., 2020). This is well exemplified for the first two variants identified in SCA42ND patients, p.A961T and p.M1531V, which localized within the intracellular gate (IG) at the IIS6 and IIIS6 segments, respectively. These variants are responsible for a slowing in the inactivation and deactivation kinetics as well as an increase in the window current (Chemin et al., 2018). Recently, several novel Ca_v_3.1 variants (p.M197R, p.V392M, p.F956del, p.I962N, p.S1263A, p.I1412T, p.G1534D and p.R1718G) were reported in patients with neurodevelopmental conditions, either fully or partially overlapping the original SCA42ND presentation (Qebibo et al., 2024). All these findings have established *CACNA1G* as involved in a broad spectrum of neurological and neurodevelopmental diseases.

Patch-clamp recordings of Ca_v_3.1 variants heterologously expressed in HEK-293 cells is a gold-standard for electrophysiological characterization of disease variants (Chemin et al., 2018). However, given the growing number of Ca_v_3.1 variants identified, conducting calcium current recordings using manual patch-clamp (MPC) appear fastidious, highlighting the need for medium/high-throughput electrophysiology for such characterization. Automated patch-clamp (APC) was recently employed for the investigation of disease-associated variants in sodium and potassium channels (Yajuan et al., 2012; Jiang et al., 2022; Vanoye et al., 2022; Ma et al., 2024). This approach was also used to investigate multiple variants associated with schizophrenia risk in the Ca_v_3.3 channel (Baez-Nieto et al., 2022). Not only can most MPC protocols be adapted for APC to ensure accurate electrophysiological characterization of recombinant Ca_v_3 channels, but APC also offers specific advantages, such as simultaneous recordings of a large number of cells (Montnach et al., 2021).

In this study, we provide the electrophysiological characterization of 18 Ca_v_3.1 variants, including 6 newly reported variants. APC and MPC approaches were used jointly to validate the experimental conditions in APC experiments, including the efficiency of transient transfection to record Ca_v_3.1 current, and the necessary adjustments of the external (5.2 mM vs. 2 mM CaCl_2_) and intracellular (CsF vs. CsCl) recording solutions. APC enabled accurate assessment of Ca_v_3.1 current density, while MPC proved more effective for studying low-expressing variants. Neuronal modeling, using the deep cerebellar neuron (DCN) framework, along with action-potential clamp experiments were further carried out to functionally classify all these variants as loss-of-function (LOF), unchanged, or gain-of-function (GOF). This analysis further confirmed the strong GOF properties of p.A961T and p.M1531V, the two recurrent variants in SCA42ND.

## Materials and methods

### Directed mutagenesis

The human *CACNA1G* complementary DNA (accession number NM_198387.2) was mutated to generate the 18 variants by using a site-directed mutagenesis service (GenScript Biotech, Netherlands). The protein variant nomenclature used here (e.g., p.A961T) is based on the UniProt protein sequence O43497 that corresponds to the full-length reference transcript, and is simplified in the Figures (A961T) for an easier reading of the panels. The plasmid expression vectors (pcDNA3-based) were then amplified to reach the necessary dilution, 6 μg/μL for transfections for the APC experiments and 1 μg/μL for transfections for the MPC experiments.

### Transient transfection

Transient transfection was performed in 35 mm Petri dishes. For MPC, HEK-293T cells were transfected using jet-PEI (QBiogen) with a 2 µg plasmid DNA mix containing 1% of a GFP-encoding construct and 99% of a Ca_v_3.1-encoding construct, either wild-type (WT) or variants channels. For APC, HEK-293 cells were transfected by electroporation using the MaxCyte STx system (MaxCyte Inc., USA). For each condition (WT and variants), 25 µg of plasmid per transfection was used. Thirty hours after transfection, cells were dissociated with Accutase, diluted and transferred into the patch-clamp apparatus. Two independent tranfections (at least) were done for each variants measured in APC experiments, and the trend was always conserved between these 2 sets of experiments (success rate, current density, biophysical parameters). WT was included in every set of experiments, to control consistency between sets of experiments and enable data normalization. Expression level and current properties were well conserved for WT among all the series of transfection.

### Automated patch clamp (APC)

APC recordings were performed using the SyncroPatch 384PE from Nanion (Munich, Germany). Whole-cell T-type currents were recorded in transiently transfected HEK-293 cells. Single-hole, 384-well recording chips were used and seeded with 300,000 cells/mL. Pulse generation and data collection were performed with the PatchControl384 v1.5.2 software (Nanion) and the Biomek v1.0 interface (Beckman Coulter). After initiating the experiment, cell catching, sealing, whole-cell formation, buffer exchanges, recording, and data acquisition were all performed sequentially and automatically. The recording solutions were purchased from Nanion. The intracellular solution contained (in mM): 10 CsCl, 110 CsF, 10 NaCl, 10 EGTA, and 10 HEPES (pH 7.2). The extracellular solution contained (in mM): 140 NaCl, 4 KCl, 1 MgCl_2_, 5 Glucose, 10 HEPES (pH 7.4) with the final concentration of CaCl_2_ adjusted to 5.2 mM. This concentration was chosen to obtain greater calcium current density and higher percentage of gigaseal recordings. Whole-cell experiments were performed at a holding potential of −100 mV at room temperature (23 °C). For APC, series resistance (Rs) was compensated by maximum 80% and/or 20 MOhm to minimize voltage errors, and capacitance was compensated using Nanion’s compensation and correction. Currents were sampled at 20 kHz. Activation and inactivation curves were built using depolarization steps lasting 3,000 ms from −120 mV to +10 mV, with 5 mV increments followed by a 200 ms depolarization step to −20 mV. Deactivation curves were built with a 20 ms pulse to −20 mV followed by 200 ms hyperpolarizing pulses from −120 mV to −60 mV. The recovery from inactivation was investigated using a double pulse protocol. Cells were first depolarized by a 1,000 ms pre-pulse at −20 mV followed by a 20–7,000 ms long interpulse interval at holding potential (−100 mV), and finally depolarized by a 100 ms test pulse at −20 mV.

### Manual clamp (MPC) and action potential (AP) clamp

Two days after transfection, cells were split at low density for whole-cell calcium current recordings using the patch-clamp technique with an Axopatch 200B amplifier (Molecular Devices). Borosilicate glass patch pipettes were used with a resistance of 1.5∼2.5 MOhm when filled with an internal solution containing (in mM): 140 CsCl, 10 EGTA, 10 HEPES, and 3 CaCl_2_ (pH adjusted to 7.25 with NaOH, ∼315 mOsm). The extracellular solution contained (in mM): 135 NaCl, 20 TEACl, 2CaCl_2_, 1 MgCl_2_ and 10 HEPES (pH adjusted to 7.25 with NaOH 1M, ∼330 mOsm). Recordings were filtered at 5 kHz. Series resistance (Rs) was compensated by 70%–80% to minimize voltage errors, and pipette and membrane capacitances were compensated using the built-in circuitry prior to data acquisition. Current traces were recorded from a holding potential (HP) of −100 mV to ensure full availability of Cav3.1 channels upon depolarization. Depolarizing test pulses (TPs) were then applied to elicit Cav3.1 currents at various membrane potentials (Vm), allowing the construction of current–voltage (I–V) relationships by plotting the maximum peak current magnitude, normalized by the cell capacitance, as a function of the voltage applied. The steady-state inactivation properties were determined using a 5-s conditioning pre-pulses ranging from −130 mV to −40 mV, followed by a test pulse (TP) at −30 mV, allowing quantification of the percentage of inactivation at each conditioning potential and determination of the half-inactivation potential (*V*_*0.5inact*_). For the action-potential clamp studies performed in MPC, the stimulation commands were (1) a regular train of spikes recorded in Purkinje neurons of the cerebellum generously provided by Dr B. P. Bean (Harvard Medical School, Boston, MA, USA) (Raman and Bean, 1997), and (2) a reticular thalamic neuron (nRT) rebound burst (Chemin et al., 2002).

### Analysis of APC and MPC recordings

The voltage–conductance relationship G_(V)_ was obtained by dividing the maximum current amplitude by the corresponding driving force with the reversal potential (V_Rev_) estimated from the intersection with the x-axis of the linear extrapolation of the last points of the I-V curve:

$$G_{\left(V\right)}=\left(I_{\text{peak}\;\left(V\right)}\right)\;/\;\left(V-V_{\text{Rev}}\right)$$

The voltage-dependent activation parameters were obtained by fitting a single Boltzmann function to the normalized conductance (G_(V)_/G_max_):

GVGmax=1/1+e V0.5act−Vm/kact

Initial values for the fitting routine (GraphPad Prism) were *G*_*max*_ = 0.01, V_Rev_ = 30 mV, *V*_*0.5act*_ = −50 mV and *k*_*act*_ = 5 mV.

Time constants (τ) for activation (τ_Act_) and inactivation (τ_Inact_) kinetics were obtained using a double-exponential fit of the current traces:

$$I=I_{\max}\;*\;\left(1-\exp\left(–t\;/\;\tau_{\text{Act}}\right)\right)\;*\;\left(\exp\left(–t\;/\;\tau_{\text{Inact}}\right)\right)+I_{\min}$$

The voltage-dependent activation parameters were obtained by fitting a single Boltzmann function with initial values: *V*_*0.5inact*_ = −70 mV, *k*_*inact*_ = 5 mV:

$$I/I_{\max}=1\;/\;\left(1+\exp\left(\left(V\;–\;V_{0.5inact}\right)\;/\;k_{inact}\right)\right)$$

The following quality control criteria were applied for APC recordings. Only cells with Rseal >300 MOhm, RSeries <18 MOhm and Ileak >−200 pA for at least 75% of the recorded sweeps within the protocol have been kept for the analyses. For activation-related protocols, were excluded cells with a slope of activation <2 mV or >9.5 mV. Similarly for inactivation-related protocols, were excluded cells with a slope of inactivation <−12 mV or >−2 mV.

### Neuronal *in silico* modeling

Modeling was performed using the NEURON simulation environment (Hines and Carnevale, 1997). The model of cerebellar nuclear neuron is based on a previously published model (Sudhakar et al., 2015), downloaded from the NEURON database at Yale University (https://modeldb.science/185513). Neuronal activities were generated using the medium value of input gain, as described previously (Sudhakar et al., 2015). The electrophysiological properties of the Ca_v_3.1 channels were modeled using Hodgkin-Huxley equations as described previously. The values obtained for the Ca_v_3.1 WT and the variant channels were substituted for the corresponding values of native T-type channels in cerebellar nuclear neurons after fitting them with the initial model values in GraphPad Prism (see equations below). The membrane voltage values were corrected for liquid junction potential, which was 4.5 mV in the recording conditions.

$$Activation\;steady\;state\;\left(\text{minf}\right)=1.0\;/\;\left(1+\exp\left(\left(v-v_{1/2}\_\text{minf}\right)/k\_\text{minf}\right)\right)$$

$$Inactivation\;steady\;state\;\left(\text{hinf}\right)=1.0\;/\;(1+\exp\left(\left(v-v_{1/2}\_\text{hinf}\right)/k\_\text{hinf}\right)$$

$$Activation\;kinetics\;\left(\text{taum}\right)=\left(C\_\text{taum}+0.333\;/\;\left(\exp\left(\left(v-v_{1/2}\_\text{taum}1\right)/\;k\_\text{taum}1\right)+\exp\left(\left(v-v_{1/2}\_\text{tau}\_m2\right)/k\_\text{taum}2\right)\right)\right)$$

$$Inactivation\;kinetics\;\left(\text{tauh}\right)=\left(C\_\text{tauh}+0.333\;/\;\exp\left(\left(v-v_{1/2}\_\text{tauh}1\right)/k\_\text{tauh}1\right)\right)$$

### Statistical analyses

Data were analyzed with GraphPad Prism and results are presented as means ± standard error of the mean (SEM). P-values for the statistical analyses were calculated using nonparametric (Kruskal–Wallis) one-way ANOVA followed by Dunnett’s *post hoc* multiple comparison test with the following significance criteria *p < 0.05, **p < 0.01, and ***p < 0.001.

## Results

### Current density measurements in APC

**FIGURE 1:**
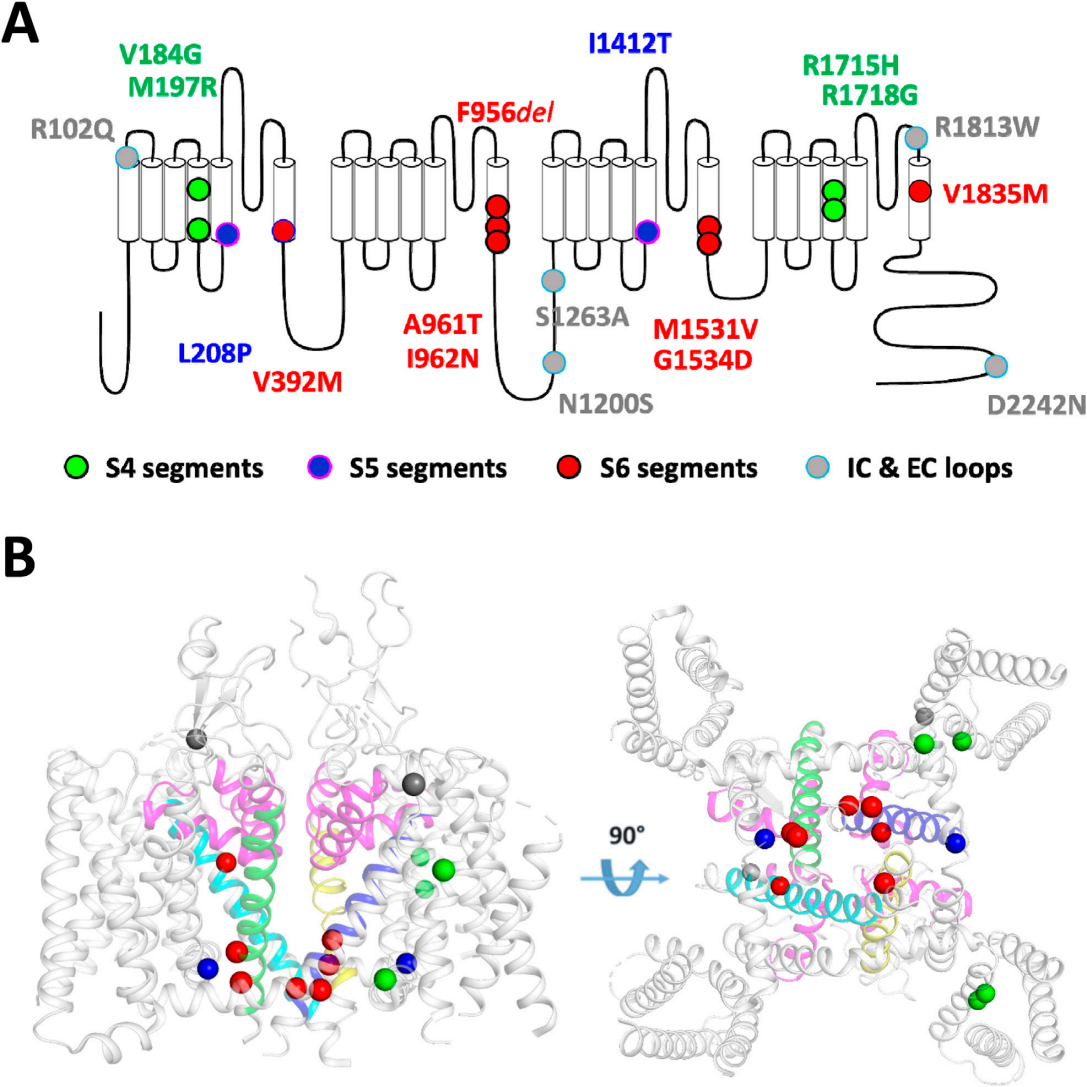
Schematic localization on the Cav3.1 channel of the 18 variants investigated in this study. **(A)** The transmembrane topology of the Cav3.1 calcium channel shows the four domains repeat (DI to DIV), each composed of six transmembrane segments (S1 to S6). Variants on segments S4, S5, S6, and the intracellular and extracellular loops (IC, EC) are depicted in green, blue, red, and gray, respectively. **(B)** 3D representation of the position of 15 variants on the Cryo-EM resolved structure of Cav3.1 (PDB: 6KZO (Zhao et al., 2019) with a side view (left panel) and a bottom view (right panel). The 3 additional variants (p.N1200S, p.S1263A and p.D2242N) are found in area of the Cav3.1 protein that were not resolved in the 6KZO PDB structure.

**FIGURE 2:**
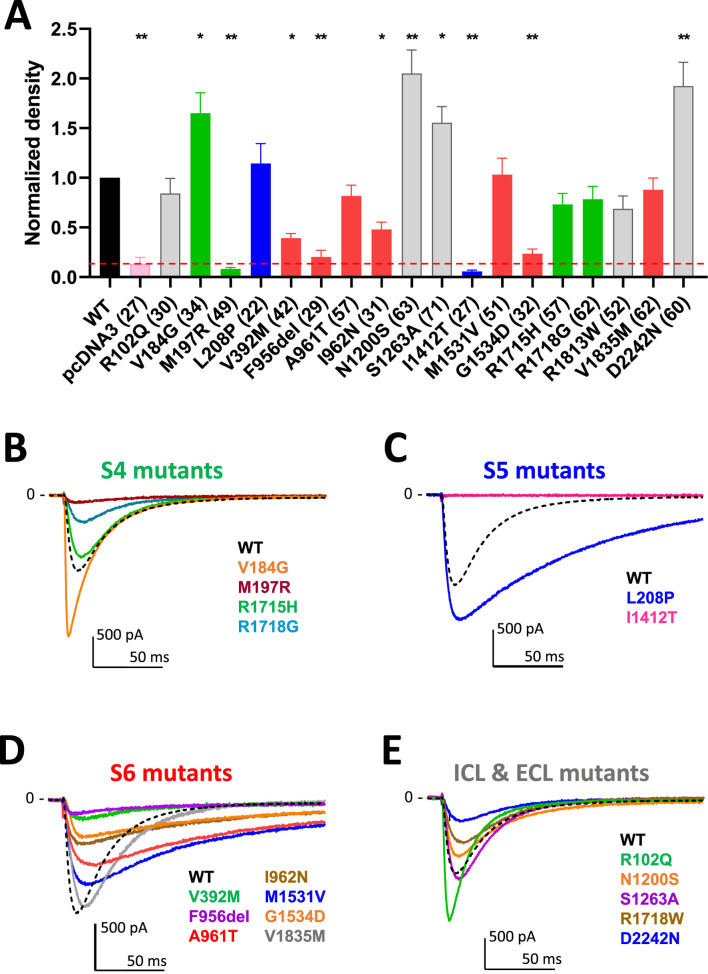
Density and calcium current traces for the 18 Cav3.1 variants studies in APC. **(A)** Graph of the calcium current density at −30 mV for all the variants, normalized to the WT current density. The red dotted line indicates the maximum density obtained on empty pcDNA3-transfected cells (∼5pA/pF) that was used to identify low-expressing cells excluded for further biophysical analysis. **(B,E)** Current trace examples at −30 mV for the all the variants grouped according to their localization in the Cav3.1 protein. Distinct colors were used to better visualize the calcium current traces illustrating each variant of the S4 group [in green, **(B)**] the S5 group [in blue, **(C)**] the S6 group [in red, **(D)**] and the group comprising variants in loops, N-ter and C-ter [in grey, **(E)**] compared to WT (black dotted line).

The 18 Ca_v_3.1 variants that we transiently transfected for electrophysiological characterization using APC are presented in Table 1 and in Figure 1. Eight variants were here investigated for the first time in electrophysiological studies: p.R102Q, p.V184G, p.N1200S, p.S1263A, p.R1718G, p.R1813W, p.V1835M, and p.D2242N. Among these, the p.S1263A and p.R1718G variants were recently reported (Riquet et al., 2023; Qebibo et al., 2024) but uncharacterized at the functional level. The six other variants were identified by the genetic diagnostic centers involved in this study. They had not previously been reported and were annotated as variants of uncertain significance (VUS). Calcium currents were recorded using MPC for 10 of these variants, p.M197R, p.L208P, p.V392M, p.F956del, p.A961T, p.I962N, p.I1412T, p.M1531V, p.G1534D, and p.R1715H in recent studies (Coutelier et al., 2015; Chemin et al., 2018; Berecki et al., 2020; Qebibo et al., 2024). Variants located on the S4 segments are shown in green, those on the S5 segments in blue, the S6 segments in red, and those on the loops in grey (Figure 1A) with 15 of them being mapped onto the cryo-EM structure of the Cav3.1 protein (Figure 1B; (Zhao et al., 2019)). In APC experiments, the percentage of Ca_v_3.1 current-positive cells was in the range of 75% (comparing Ca_v_3.1 WT transfected cells with mock transfected cells). Only cells with a current density greater than 5pA/pF were considered for further calcium current analyses. This approach led us identify 6 variants (p.M197R, p.V392M, p.F956del, p.I962N, p.I1412T, and p.G1534D) with current densities significantly lower than Ca_v_3.1 WT channels (Figure 2A; Supplementary Table S1; Supplementary Figures S1–S8). Four of these variants, p.M197R, p.F956del, p.I1412T, and p.G1534D, previously characterized using MPC (Qebibo et al., 2024), exhibited too few cells with sufficiently large enough calcium current density for accurate biophysical characterization in APC experiments. These 4 variants, along with p.V392M, for which only half-activation potentials could be determined, were excluded from further analysis of their APC recordings. Only the p.I962N variant exhibited a sufficiently high average current density and quality current traces for complete APC-based characterization. Contrasting with low expressing variants, p.V184G, p.N1200S, p.S1263A and p.D2242N variants displayed significantly higher current densities (Figure 2A). The superimposed representative current traces at −20 mV (Figures 2B–E) illustrate the difference in current density across all these variants, especially those with near-null current density (p.M197R and p.I1412T). In addition, these current traces revealed the pronounced differences in inactivation kinetics, which appeared markedly slowed for the variants located on the S5 and S6 segments defining the IG (Figures 2C,D).

**TABLE 1:** Presentation of the 18 *CACNA1G* variants investigated in the study in automated patch-clamp (APC), manual patch-clamp (MPC) or both (APC/MPC). The clinical description of the variants in black can be found in previous studies (see references).

Variants	MPC/APC tested	Inheritance	Clinical information
**p.R102Q**	APC	*not known*	Ataxia, progressive cerebellar atrophy, global developmental delay. Medical history complicated by prenatal exposures to drugs/alcohol. Possible encephalitis in infancy. Variant also present in EXAC.
**p.V184G**	APC	*not known*	Adult-onset neuromuscular disease, including ptosis, muscle weakness, peripheral neuropathy, and ataxia
p.M197R	APC/**MPC**	*de novo*	Qebibo et al. (2024)
p.L208P	APC	*de novo*	Berecki et al. (2020)
p.V392M	APC/MPC	*de novo*	Qebibo et al. (2024)
p.F956del	APC/**MPC**	*de novo*	Qebibo et al. (2024)
p.A961T	APC/MPC	*de novo*	Chemin et al. (2018), Qebibo et al. (2024)
p.I962N	APC/MPC	*de novo*	Qebibo et al. (2024)
**p.N1200S**	APC/MPC	*de novo*	Kosmicki et al. (2017)
p.S1263A	APC	*de novo*	Qebibo et al. (2024)
p.I1412T	APC/**MPC**	*de novo*	Qebibo et al. (2024)
p.M1531V	APC/MPC	*de novo*	Chemin et al. (2018), Qebibo et al. (2024)
p.G1534D	APC/**MPC**	*de novo*	Qebibo et al. (2024)
p.R1715H	APC/MPC	inherited	Coutelier et al. (2015)
p.R1718G	APC	*de novo*	Riquet et al. (2023), Qebibo et al. (2024)
**p.R1813W**	APC		Congenital ataxia. Found in one patient, inherited from his mother, both also having a known pathogenic *CACNA1A* variant but with incomplete penetrance/expressivity
**p.V1835M**	APC/MPC	inherited	A 2.5-year-old girl (in 2019) with developmental delay, microcephaly, and tremor (when scared/anxious). Exome sequencing revealed a pathogenic variant in *MECP2*, so she has Rett syndrome. She also has tremor. Her brain MRI at 1.5 years was normal; it did not show cerebellar atrophy
**p.D2242N**	APC	*not known*	Mild intellectual disability, ophthalmoplegia, and progressive ataxia, dysarthria, and dysphagia. He has developed prognathism. Brain MRI shows olivopontocerebellar atrophy

The variants in bold-red are reported for the first time, with a brief clinical description of the related patients. Bold-underlined MPC indicates that the properties of these variants were preferentially obtained in MPC.

### Biophysical properties of variants characterized by APC

**FIGURE 3:**
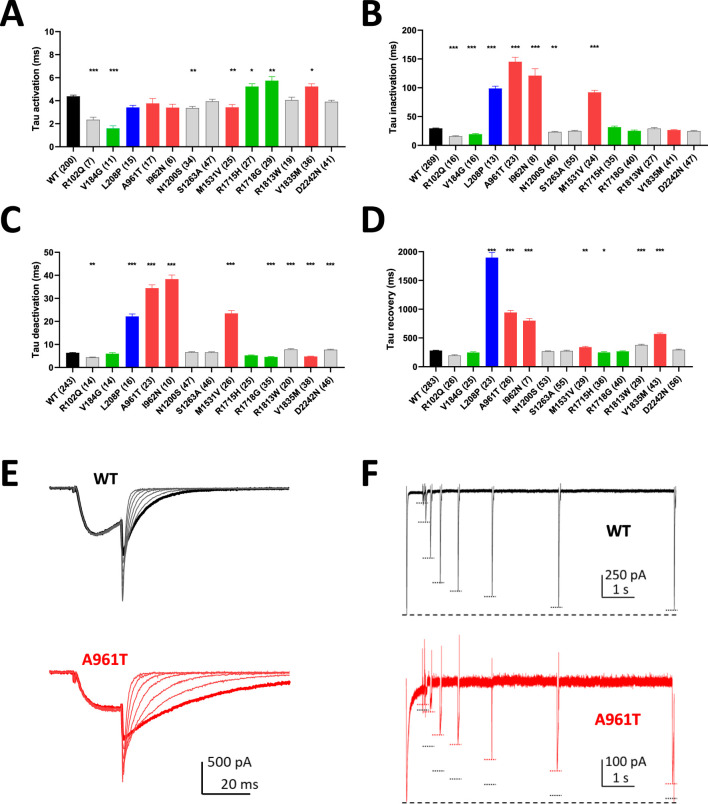
Kinetic properties of Cav3.1 variants using APC. **(A)** Activation kinetics at −20 mV. **(B)** Inactivation kinetics at −20 mV. **(C)** Deactivation kinetics at −60 mV. **(D)** Recovery from inactivation. **(E)** Examples of APC recordings of deactivation kinetics for WT (black) and p.A961T (red) with bold traces corresponding to the repolarization step at −60 mV. **(F)** Examples of APC recordings of the recovery from inactivation for WT (black) and p.A961T (red). Both in the upper and lower graphs, the large dotted lines at the bottom correspond to P1 amplitude and every P2 amplitude is marked with a short dotted line (black for WT and red for p.A961T). Note that the recordings for the variants p.M197R, p.V392M, p.F956del, p.I1412T and p.G1534D did not meet quality controls for measuring all the electrophysiological parameters. Variants on segments S4, S5, S6, and IC and EC loops are represented in green, blue, red, and gray, respectively.

**FIGURE 4:**
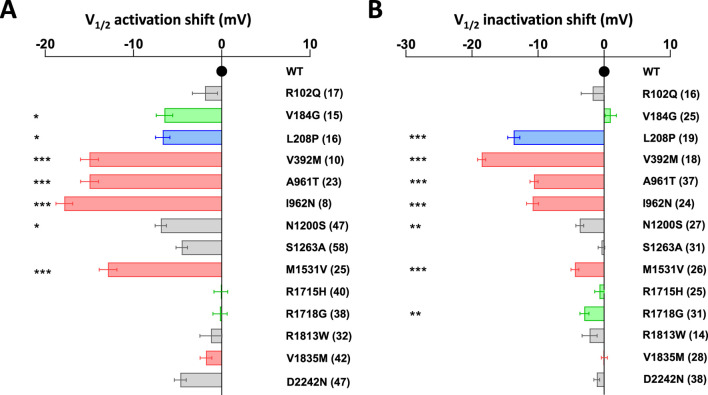
Half-activation and inactivation properties of Cav3.1 variants using APC. **(A)** The shift in steady-state half-activation potential (V_1/2_ activation) for each variant is compared to the WT value. **(B)** The shift in steady-state half-inactivation potential (V_1/2_ inactivation) for each variant is compared to the WT value. Variants on segments S4, S5, S6, and IC and EC loops are represented in green, blue, red, and gray, respectively.

The activation kinetics is rather fast for the WT Ca_v_3.1 current (∼4 ms at −20 mV) in APC recordings. The S4 variants p.R1715H and p.R1718G, as well as the S6 variant p.V1835M, exhibit slower activation, while most of the variants in S5 and S6 segments display faster activation (Figure 3A; Supplementary Figure S1; Supplementary Table S1), compared to WT. Similar to findings reported using MPC, the inactivation and deactivation kinetics are significantly slower for the S5 and S6 IG variants (p.L208P, p.A961T, p.I962N, p.M1531V) (Figures 3B,C; Supplementary Figures S2, S3; Supplementary Table S1). Regarding recovery from inactivation, the p.L208P, p.A961T, and p.I962N variants exhibit the slowest recovery rates and do not fully recover from inactivation (Figure 3D; Supplementary Figure S4; Supplementary Table S1), again in good agreement with MPC experiments. Examples of current traces illustrating APC recordings of deactivation kinetics and recovery from inactivation are provided for WT and p.A961T variant (Figures 3E,F). Notably, all the variants evaluated in APC experiments display no shift or a negative shift in their steady-state activation and inactivation properties (Figures 4A,B; Supplementary Table S1). Consistent with MPC experiments, these hyperpolarizing shifts were highly significant for the IG variants p.L208P, p.V392M, p.A961T, p.I962N and p.M1531V.

### Correlation between APC and MPC data

**FIGURE 5:**
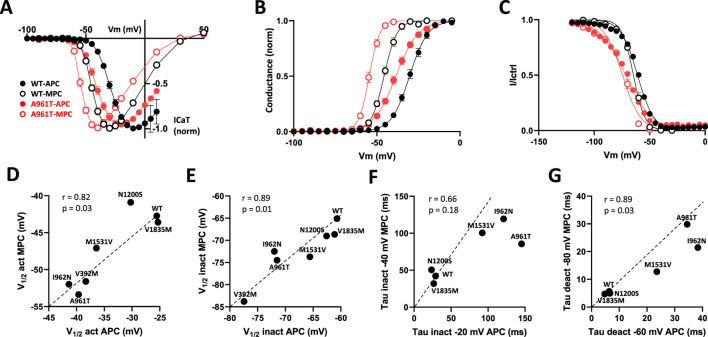
Comparison of MPC and APC electrophysiological parameters. **(A)** I-V curves obtained using MPC (open symbols) and APC (filled symbols) for WT (black) and the recurrent SCA42ND variant p.A961T (red). **(B)** Steady-state activation curves. **(C)** Steady-state inactivation curves. **(D–G)** The correlation graphs for half-activation potential **(D)** half-inactivation potential **(E)** inactivation kinetics **(F)** and deactivation kinetics **(G)** respectively, measured in APC (X axis) and MPC (Y axis). Variants on segments S4, S5, S6, and IC and EC loops are represented in green, blue, red, and gray, respectively.

Superimposed current-voltage relationships (Figure 5A), steady-state activation (Figure 5B) and steady-state inactivation (Figure 5C) recorded in MPC and APC for the WT and the most recurrent SCA42ND variant (p.A961T) clearly illustrate that the hyperpolarizing shift in steady-state properties for the p.A961T variant was comparable in MPC and APC experiments. The use of a higher extracellular calcium concentration in APC (5.2 mM), compared to MPC (2 mM), resulted in a depolarizing shift of similar amplitude for both the WT and p.A961T variant in steady-state activation (15 mV for WT and 16 mV for p.A961T) and in steady-state inactivation (4 mV for WT and 3 mV for p.A961T). We then assessed the correlation between the electrophysiological properties measured in APC and MPC for the set of variants studied in both experiments (Figures 5D–G). Indeed, both steady-state activation (V_1/2act_) and steady-state inactivation (V_1/2inact_) showed a robust correlation (r close to 1, p < 0.05) between APC and MPC (Figures 5D,E), as well as for deactivation kinetics and recovery from inactivation (Figure 5G; Supplementary Figure S5). However, no correlation was observed for inactivation kinetics data (r = 0.66), likely due to the dispersion of values for slow-inactivating variants, compared to the highly clustered WT-like variants (Figure 5F). A lack of correlation was also observed for the activation kinetics (Supplementary Figure S5).

### Predicted consequence of Ca_v_3.1 variants properties on neuronal excitability

**FIGURE 6:**
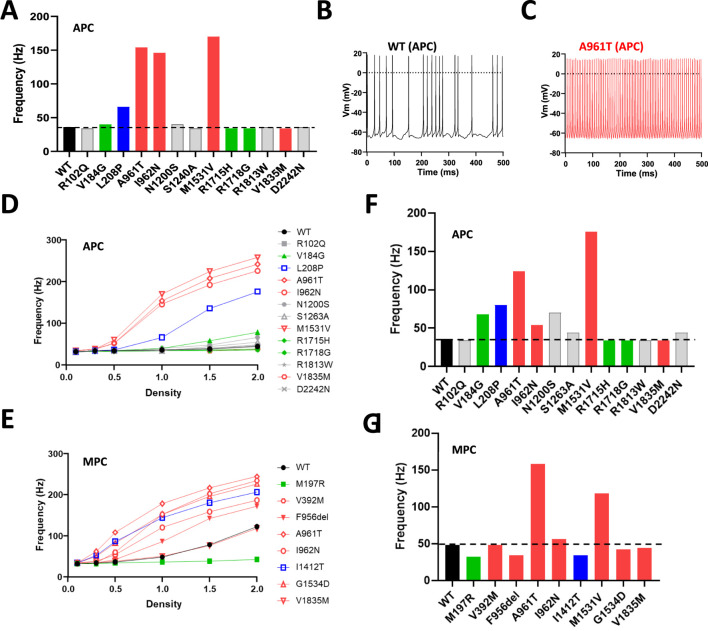
Deep Cerebellar Nucleus (DCN) modeling using Cav3.1 variant parameters obtained in APC and in MPC experiments. **(A)** Spike frequency graph for Cav3.1 variants characterized using APC. **(B,C)** Examples of DCN spiking activity for using APC parameters obtained for WT **(B)** and p.A961T channels **(C)**. **(D,E)** Change in DCN spike frequency obtained with increasing current densities for all the variants characterized in APC **(D)** and in MPC **(E)** respectively. **(F,G)** The DCN spike frequency obtained for the current density measured in Figure 2B (normalized to the WT current density) for the variants characterized in APC **(F)** and MPC **(G)** respectively.

The electrophysiological parameters collected in MPC and APC experiments (V_1/2_ act, V_1/2_ inact, Kact, Kinact, Tau act, Tau inact, Tau deact) were used in a virtual model of DCN neurons (Sudhakar et al., 2015) to estimate the effects of the variants on DCN firing activity by measuring the action potential (AP) frequency (Figure 6; Supplementary Figures S6-S8). We recently reported that the IG variants investigated in MPC (p.V392M, p.F956del, p.A961T, p.I962N, p.I1412T, p.M1531V, p.G1534D) produced a higher AP frequency compared to WT (Qebibo et al., 2024). Notably, using the parameters of the variants fully explored with the APC approach, the p.A961T, p.I962N and p.M1531V variants, and to a lesser extent the p.L208P variant, also showed a higher AP frequency (Figure 6A) as exemplified for p.A961T compared to WT (Figures 6B,C). Since APC experiments revealed that Ca_v_3.1 variants exhibit either increased or decreased current density compared to WT channel (Figure 2A), we next investigated the impact of varying current density on AP frequency for all the variants studied in APC (Figure 6D) and those studied in MPC (Figure 6E). A 2-fold increase in current density resulted in a marked AP-frequency increase for GOF variants, especially p.L208P (Figure 6D). WT-like variants also displayed increased AP-frequency, while no change was observed for the loss-of-channel-activity variant p.M197R (Figure 6E). Finally, when DCN modeling was performed using the Ca_v_3.1 current density measured in APC (see Figure 2A), only the IG variants p.M1531V > p.A961T > p.L208P > p.I962N, as well as the variants p.V184G and p.N1200S showed increased AP-frequency (Figures 6F,G; Supplementary Figures S7-S8).

### GOF properties in action-potential clamp experiments for p.M1531V and p.A961T

**FIGURE 7:**
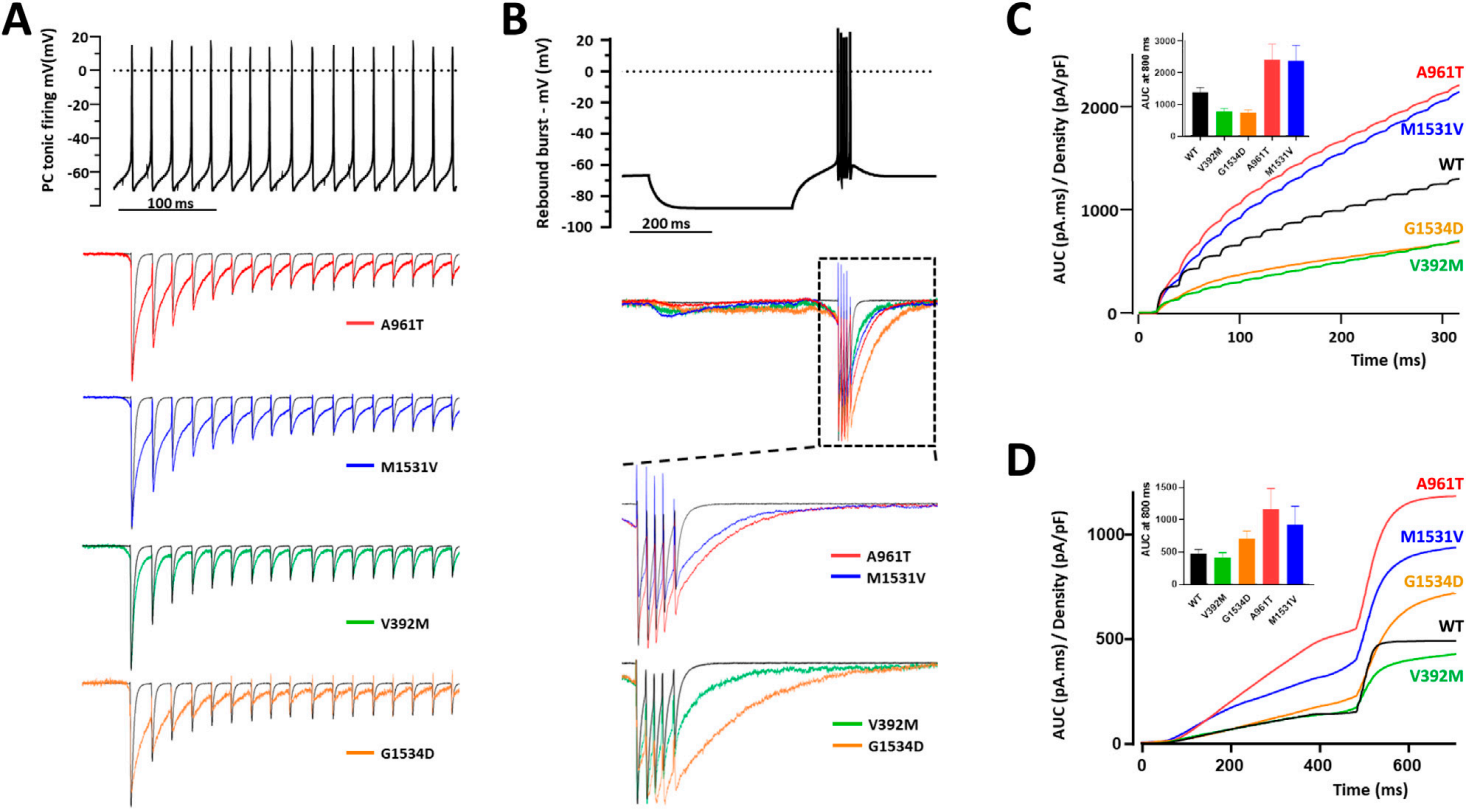
Action-potential clamp experiments with IG variants. **(A)** Representative calcium current traces, normalized to the 1st AP, for p.A961T, p.M1531V, p.V392M, and p.G1534D variants (lower panel) in response to a Purkinje cell tonic firing activity used as voltage command (upper panel). **(B)** Representative calcium current traces, normalized to the 1st AP, for p.A961T, p.M1531V, p.V392M, and p.G1534D variants (lower panel) in response to a thalamic rebound burst firing activity used as voltage command (upper panel). **(C,D)** The integral (area under the curve) quantification of the calcium current for each variant obtained for the tonic firing activity **(C)** and the rebound burst activity **(D)** respectively. The histograms of the average AUC values at 800 ms (endpoint of the recordings) are shown as insets in panels 7C and 7D.

A further functional evaluation of the GOF properties of four representative IG variants (p.V392M, p.A961T, p.M1531V, p.G1534D) was performed using action potential (AP) clamp experiments in MPC (Figure 7). Calcium currents were recorded in HEK-293 cells expressing these variants during an AP-voltage command, mimicking (i) a tonic firing activity of Purkinje cells and (ii) a rebound burst firing from thalamic nRT neurons. In these recordings, the resulting calcium current reflects the specific electrophysiological behavior of each variant (Figures 7A,B). For tonic firing activity, it is noteworthy that most of the inward current occurred during the interspike interval, linked to the slow deactivation of Ca_v_3.1, which was even slower for IG variants (Figure 7A). For rebound burst firing activity, the calcium current relies on both Ca_v_3.1 de-inactivation and slow deactivation (Figure 7B). The area under the curve (AUC) was measured and normalized to the maximum amplitude of the current density recorded in each cell using a standard test-pulse protocol. The p.A961T and p.M1531V variants exhibited a significant increase in calcium current during both tonic and rebound burst activities (Figures 7C,D). Conversely, the p.V392M and p.G1534D variants showed reduced calcium current during tonic firing activity (Figure 7C). During rebound burst activity, the p.G1534D variant exhibited a moderate increase in calcium current, whereas the p.V392M variant showed lower calcium entry, compared to WT (Figure 7D). AP waveform rebound burst model confirms slowed deactivation of all the variants and shows an increased activity at step to hyperpolarizing voltages for variants with GOF features (A961T and M1531V) but not for variants with mix GoF and LoF features (e.g., V392M and G1534D). These AP clamp experiments highlight that the Ca_v_3.1 variant-dependent calcium entry is influenced by the neurons’ electrophysiological behavior and further confirm the GOF properties of the p.A961T and p.M1531V variants.

## Discussion

### Automated patch-clamp techniques for classification of Cav3 channelopathies

The APC technology is revolutionizing the field of electrophysiology, including for the study of channelopathies, by allowing investigation of a large number of variants associated with various neurological and cardiac disorders (Vanoye et al., 2021). APC allows to record and analyze hundreds of cells in parallel, providing a more comprehensive characterization of ion channel variants (Ng et al., 2021; Ma et al., 2023). Traditional MPC techniques are highly accurate in describing parameters, but time-consuming and labor-intensive, whereas APC offers high-throughput capability and may be less operator-dependent (Yajuan et al., 2012). This is particularly crucial given the growing number of identified ion channel variants (Lukacs et al., 2021; Obergrussberger et al., 2022) as evidenced here with the discovery of many novel *CACNA1G* variants associated to neurological conditions. We and others have shown that APC experiments using the SyncroPatch PE384 are reliable in transposing electrophysiological parameters to study voltage-gated sodium and potassium channels, provided that appropriate guidelines are followed (Glazer et al., 2020; Montnach et al., 2021; Oliveira-Mendes et al., 2023). T-type Ca_v_3 calcium channels are particularly well suited for APC investigations as only the pore channel protein (Ca_v_α_1_) is required to produce a native-like T-type calcium current (Chemin et al., 2002; Perez-Reyes, 2003). Indeed, APC was recently used to study 57 variants of the Ca_v_3.3 channel identified in a large schizophrenia cohort (Baez-Nieto et al., 2022). Here, we have also successfully used APC to characterize 18 Ca_v_3.1 variants found in patients with neurological phenotype, especially 8 VUS (p.R102Q, p.V184G, p.N1200S, p.S1263A, p.R1718G, p.R1813W, p.V1835M, and p.D2242N) that are being studied at the functional level for the first time. Our study validates that MPC experimental conditions could be adequately transposed to APC for Ca_v_3.1 study. Importantly, the gating defects observed for the *de novo* IG variants (S5-S6 segments) using MPC, i.e., slow inactivation and deactivation kinetics, hyperpolarizing shift of steady-state activation and inactivation properties (Chemin et al., 2018; Berecki et al., 2020; Qebibo et al., 2024) were accurately replicated in APC experiments (Figures 2, 3). This was also validated for the other variants studied in both MPC and APC experiments. APC offers the advantage of recording a large number of cells, blindly, for each condition (variant), which is well-suited for measuring the variant current density. While some variants displayed increased current density, our study also points to a few variants showing very low current density. The surface expression and/or stability at the plasma membrane might be affected for some of these variants resulting in reduced, or increased, current densities. Possibly, substitution of Met197 with Arg may result in a constrained flexibility of the intracellular S4-S5 linker and altered gating (with reduced current). Further studies will be needed to investigate all the potential mechanisms underlying alteration in the current density. According to our quality control criteria, low expressing (LOF) variants resulted in a small number of cells that could be accurately studied in APC. The difficulty in studying LOF variants may be a caveat when examining large series of variants using APC only. Increasing the number of APC recordings for variants with small currents should favor their analysis to a standard similar to that achieved using MPC. In addition, to better assess how the data correlate between in MPC and APC experiments, future studies should study the impact of some of the specific requirements in APC recording conditions, e.g., the concentration of 5.2 mM external calcium, on the negative shift of activation and inactivation, as well as on the kinetics of activation and inactivation considering that external calcium concentration has been shown to influence Cav3 channel gating (Lacinova et al., 2006; Cazade et al., 2017). Overall, the APC and MPC approaches were highly complementary in providing a comprehensive electrophysiological analysis of our large series of Ca_v_3.1 variants.

### Deciphering the gain/loss of channel activity in support of GOF or LOF variants

Several GOF variants in *CACNA1G* (*de novo*, missense mutations) are now linked to a variety of neurological and neurodevelopmental diseases with some severe conditions such as SCA42ND (Chemin et al., 2018; Qebibo et al., 2024). Deciphering the electrophysiological alterations caused by these mutations in the Ca_v_3.1 channels is necessary to better document the disease mechanism(s) and identify potential therapeutic opportunities. The electrophysiological criteria supporting a gain of channel activity are the increase in current density, the hyperpolarizing shift of the steady-state activation curve, the slower inactivation and deactivation kinetics and the increased window current (Chemin et al., 2018; Berecki et al., 2020; Qebibo et al., 2024). In turn, lower current density, slower recovery from inactivation, and a hyperpolarized steady-state inactivation curve are indicative of loss of channel activity (Chemin et al., 2018; Qebibo et al., 2024). To date, most analyses of the channel variants’ gain/loss of channel activity are performed in heterologous expression systems, e.g., in transfected HEK-293 cells (as here), without considering some specificities of the native distribution of the studied channel. Recently, neuronal modeling was used to support MPC findings for several Cav3 variants and mutants (Blesneac et al., 2015; Coutelier et al., 2015; Chemin et al., 2018; El Ghaleb et al., 2021; Baez-Nieto et al., 2022; Qebibo et al., 2024). Ca_v_3.1 is highly expressed in several cerebellar neurons, especially in the deep cerebellar nucleus (DCN), for which virtual neuron models have been developed (Destexhe et al., 1996; Anwar et al., 2012; Sudhakar et al., 2015). We show here that the use of computed neuronal excitability (Sudhakar et al., 2015) allowed us to pinpoint the gain/loss of channel properties for the eighteen variants explored in this study either in MPC, APC or both. Coupling APC and MPC data with *in silico* neuronal modeling appears to be a robust and complementary method for classifying Ca_v_3.1 variants. This approach further described the two recurrent SCA42ND variants p.A961T and p.M1531V as GOF variants.

In this study, we also report that the ‘gain/loss of channel activity’ toolbox could be completed with action potential (AP) clamp experiments. AP clamp experiments were performed in HEK-293 cells with neuronal activities originating from cells known to express Ca_v_3.1 (cerebellar and thalamic neurons) as voltage commands (Chemin et al., 2002). The GOF ability of the two SCA42ND variants p.A961T and p.M1531V was retrieved both using tonic firing (Purkinje neuron) and rebound burst firing (thalamic neuron). The two other IG variants examined here, p.V392M and p.G1534D, both displayed reduced activity with tonic firing voltage command, while p.G1534D, but not p.V392M, showed increased calcium entry in rebound burst firing. Overall, *in silico* modeling and AP clamp experiments revealed that at the functional level, the GOF properties of the variants are intimately associated with the specific electrophysiological signature of cells expressing the Ca_v_3.1 channel. Our study demonstrates that these experiments add to the variant characterization pipeline by contributing to a better classification of channel variants. To characterize further the GOF/LOF properties of *CACNA1G* variants, *in vivo* studies will also be instrumental. However, this will require the development of appropriate models (human iPSC-derived cellular models or animal models) to take into account the diversity of channel variants, while complying with the 3R rule (Diaz et al., 2020).

## Limitations of the study and perspectives

In the present study, the main Cav3.1 variant-specific electrophysiological properties (i.e., inactivation kinetics and voltage-dependence of activation and inactivation) were successfully described using APC. In future studies, APC protocols, adapted here from standard MPC protocols should be further optimized to enable a more in-depth and accurate analysis of Cav3 variants, towards improving the classification of these variants. One limitation of our APC approach was in best characterizing some of the current properties (peak conductance, V_Rev_) for variants showing low current amplitude or low percentage of positive cells. This issue could be overcome by recording a larger number of cells in transient transfection assays or by creating high-expressing cell lines for each variant. The use of 5.2 mM external Ca^2+^ concentration, a recommended technical guideline for our APC platform, may also be modified either to a more physiological external Ca^2+^ concentration (∼2 mM) or to a higher external Ca^2+^ concentration (10 mM). Higher external Ca^2+^ would expectedly favor a more accurate V_Rev_ determination for improved current-voltage fitting and overall adjustment of the APC quality control criteria, enabling also the study of any potential changes in Ca^2+^ modulation (permeation, facilitation) among variants. A similar validation process may also be necessary when setting up biophysical analyses of channel variants with other APC platforms. While MPC remains valuable for in-depth analysis, variant classification should greatly benefit from every improvement made in APC experiments in future studies.

## Data Availability

The raw data supporting the conclusions of this article will be made available by the authors, without undue reservation.
