# Supplementary material for: Electrophysiological classification of CACNA1G gene variants associated with neurodevelopmental and neurological disorders
Source: Front Pharmacol. 2025 Oct 2;16:1613072. doi: 10.3389/fphar.2025.1613072 (PMC12528054; doi:10.3389/fphar.2025.1613072)
Supplement: Supplementary file 1 [file DataSheet1.pdf]

	Cav3.1 WT	Cav3.1 p.R102Q	Cav3.1 p.V184G	Cav3.1 p.M197R
**Normalized Current density to WT at -30 mV**	1 ± 0.13 (389)	0.84 ± 0.15 (30)	1.65 ± 0.21 (34)*	0.08 ± 0.01 (49)**
**V0.5 activation shift from WT (mV)**	0 ± 0.19 (219)	-1.89 ± 1.49 (17)	-6.48 ± 0.93 (15)*	-
**V0.5 inactivation shift from WT (mV)**	0 ± 0.11 (98)	-1.71 ± 1.76 (16)	-1.01 ± 0.84 (25)	-
**Activation kinetics at -20 mV (Tau act, ms)**	4.39 ± 0.10 (200)	2.35 ± 0.22 (7)***	1.61 ± 0.22 (11)***	-
**Inactivation kinetics at -20mV (Tau inact, ms)**	29.54 ± 0.63 (269)	15.87 ± 0.82 (16)***	19.42 ± 1.13 (16)***	-
**Deactivation kinetics at -60 mV (Tau deact, ms)**	6.38 ± 0.16 (243)	4.41 ± 0.15 (14)**	6.01 ± 0.48 (14)	-
**Recovery (Tau recov, ms)**	282.40 ± 4.59 (283)	195.39 ± 11.38 (26)	247.44 ± 11.26 (25)	-

	Cav3.1 p.L208P	Cav3.1 p.V392M	Cav3.1 p.F956del	Cav3.1 p.A961T
**Normalized Current density to WT at -30 mV**	1.14 ± 0.20 (22)	0.39 ± 0.04 (42)*	0.20 ± 0.06 (29)**	0.82 ± 0.11 (57)
**V0.5 activation shift from WT (mV)**	-6.67 ± 0.81 (16)*	-14.97 ± 1.00 (10)***	-	-14.96 ± 1.01 (23)***
**V0.5 inactivation shift from WT (mV)**	-13.67 ± 0.93 (19)***	-18.53 ± 0.61 (18)***	-	-10.60 ± 0.61 (37)***
**Activation kinetics at -20 mV (Tau act, ms)**	3.42 ± 0.17 (15)	-	-	3.77 ± 0.41 (17)
**Inactivation kinetics at -20mV (Tau inact, ms)**	98.88 ± 4.08 (13)***	-	-	145.25 ± 7.68 (23)***
**Deactivation kinetics at -60 mV (Tau deact, ms)**	22.18 ± 1.03 (16)***	-	-	34.48 ± 1.42 (23)***
**Recovery (Tau recov, ms)**	1897.30 ± 88.14 (23)***	-	-	943.13 ± 34.25 (26)***

	Cav3.1 p.I962N	Cav3.1 p.N1200S	Cav3.1 p.S1263A	Cav3.1 p.I1412T
**Normalized Current density to WT at -30 mV**	0.48 ± 0.07 (31)*	2.05 ± 0.23 (63)**	1.55 ± 0.16 (71)*	0.06 ± 0.01 (27)**
**V0.5 activation shift from WT (mV)**	-17.82 ± 0.95 (8)***	-6.90 ± 0.64 (47)*	-4.52 ± 0.64 (58)	-
**V0.5 inactivation shift from WT (mV)**	-10.82 ± 0.90 (24)***	-1.89 ± 0.60 (27)**	-0.39 ± 0.50 (31)	-
**Activation kinetics at -20 mV (Tau act, ms)**	3.41 ± 0.28 (6)	3.36 ± 0.14 (34)**	3.95 ± 0.18 (47)	-
**Inactivation kinetics at -20mV (Tau inact, ms)**	121.03 ± 12.10 (8)***	23.58 ± 0.86 (46)**	25.00 ± 0.88 (55)	-
**Deactivation kinetics at -60 mV (Tau deact, ms)**	38.34 ± 1.82 (10)***	6.64 ± 0.28 (47)	6.61 ± 0.20 (46)	-
**Recovery (Tau recov, ms)**	800.33 ± 38.64 (7)***	269.14 ± 7.83 (53)	275.59 ± 11.15 (55)	-

	Cav3.1 p.M1531V	Cav3.1 p.G1534D	Cav3.1 p.R1715H	Cav3.1 p.R1718G
**Normalized Current density to WT at -30 mV**	1.03 ± 0.16 (51)	0.24 ± 0.04 (32)**	0.73 ± 0.11 (57)	0.78 ± 0.13 (52)
**V0.5 activation shift from WT (mV)**	-12.87 ± 1.00 (25)***	-	-0.09 ± 0.77 (40)	-0.18 ± 0.81 (38)
**V0.5 inactivation shift from WT (mV)**	-4.42 ± 0.60 (26)***	-	-0.69 ± 0.71 (25)	-2.98 ± 0.68 (31)**
**Activation kinetics at -20 mV (Tau act, ms)**	3.43 ± 0.25 (25)**	-	5.24 ± 0.24 (27)*	5.75 ± 0.35 (29)**
**Inactivation kinetics at -20mV (Tau inact, ms)**	92.15 ± 3.42 (24)***	-	31.66 ± 1.77 (35)	25.33 ± 1.20 (40)
**Deactivation kinetics at -60 mV (Tau deact, ms)**	23.54 ± 1.18 (26)***	-	5.27 ± 0.15 (25)	4.61 ± 0.10 (35)***
**Recovery (Tau recov, ms)**	340.80 ± 12.98 (29)**	-	249.50 ± 12.48 (36)*	268.33 ± 9.06 (40)

	Cav3.1 p.R1813W	Cav3.1 p.V1835M	Cav3.1 p.D2242N
**Normalized Current density to WT at -30 mV**	0.68 ± 0.13 (52)	0.88 ± 0.12 (62)	1.92 ± 0.24 (60)**
**V0.5 activation shift from WT (mV)**	-1.21 ± 1.25 (32)	-1.78 ± 0.66 (42)	-4.68 ± 0.67 (47)
**V0.5 inactivation shift from WT (mV)**	-2.19 ± 1.13 (14)	-0.03 ± 0.47 (28)	-1.11 ± 0.43 (38)
**Activation kinetics at -20 mV (Tau act, ms)**	4.06 ± 0.24 (19)	5.23 ± 0.23 (36)*	3.91 ± 0.14 (41)
**Inactivation kinetics at -20mV (Tau inact, ms)**	29.26 ± 1.59 (27)	26.50 ± 1.13 (41)	24.98 ± 0.80 (47)
**Deactivation kinetics at -60 mV (Tau deact, ms)**	7.83 ± 0.34 (20)***	4.79 ± 0.15 (38)***	7.71 ± 0.24 (46)***
**Recovery (Tau recov, ms)**	379.24 ± 12.24 (29)***	570.27 ± 16.62 (43)***	296.22 ± 9.54 (56)

Values are presented as Mean ± SEM (n = number of cells)
**p* < 0.05 ; ***p* < 0.01 ; ****p* < 0.001 compared to Wild Type (WT) channels using non parametric (Kruskall-Wallis) One way ANOVA followed by Dunnet’s multiple comparison post hoc test. p values < 0.05 were considered statistically significant.

Supplementary Table S1

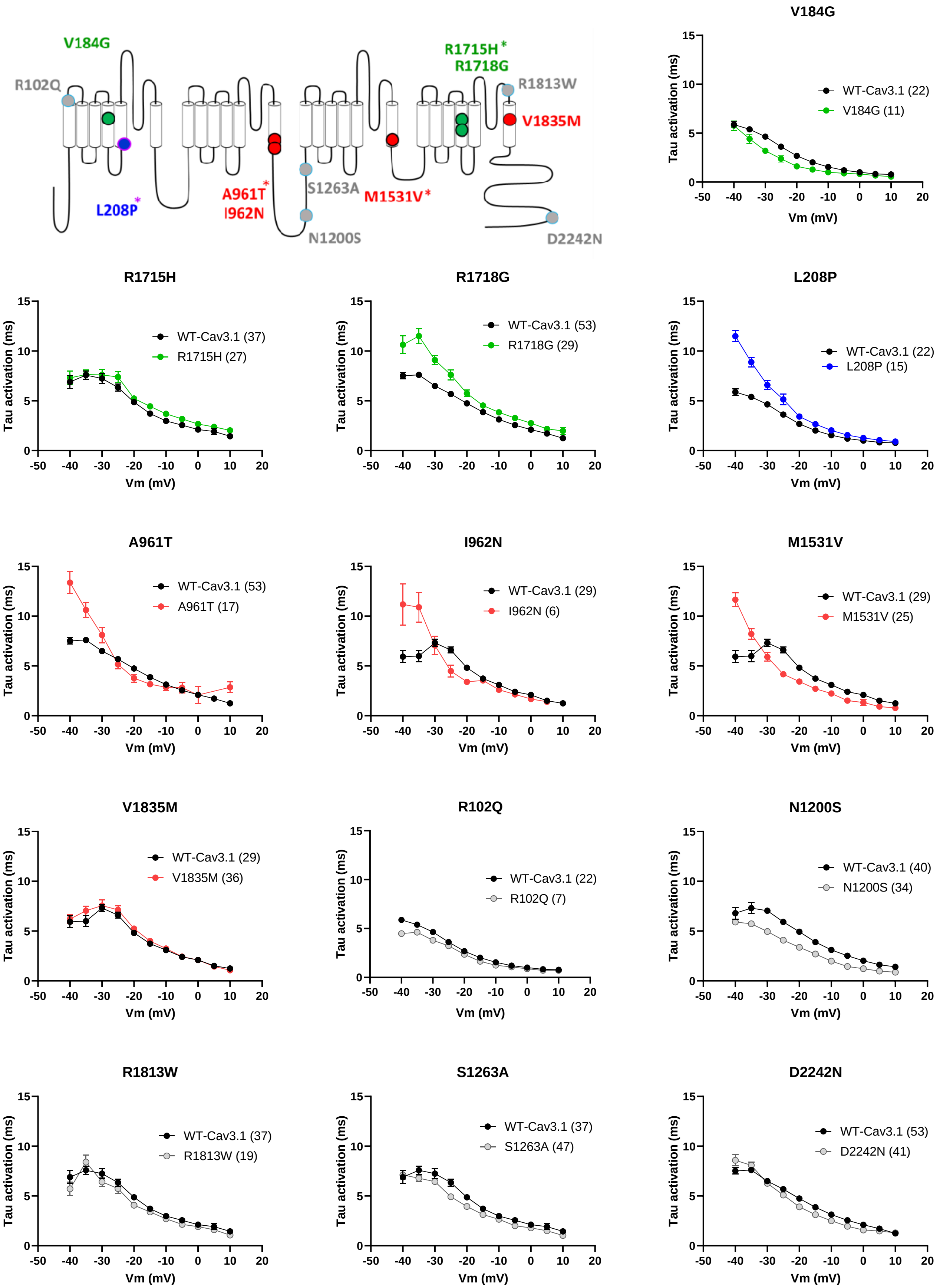

Supplementary Figure S1 : *activation kinetics in APC*

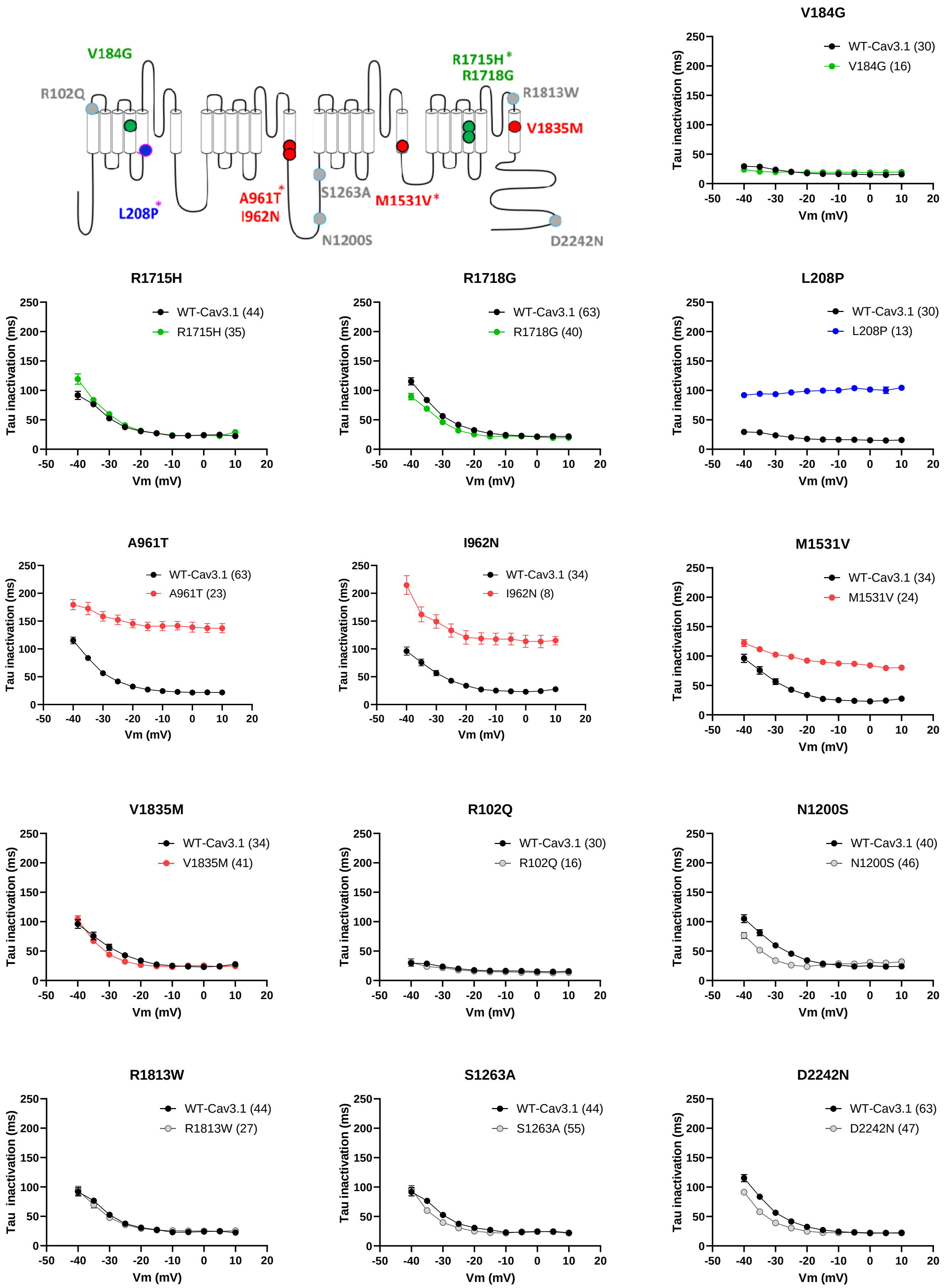

Supplementary Figure S2 : *inactivation kinetics in APC*

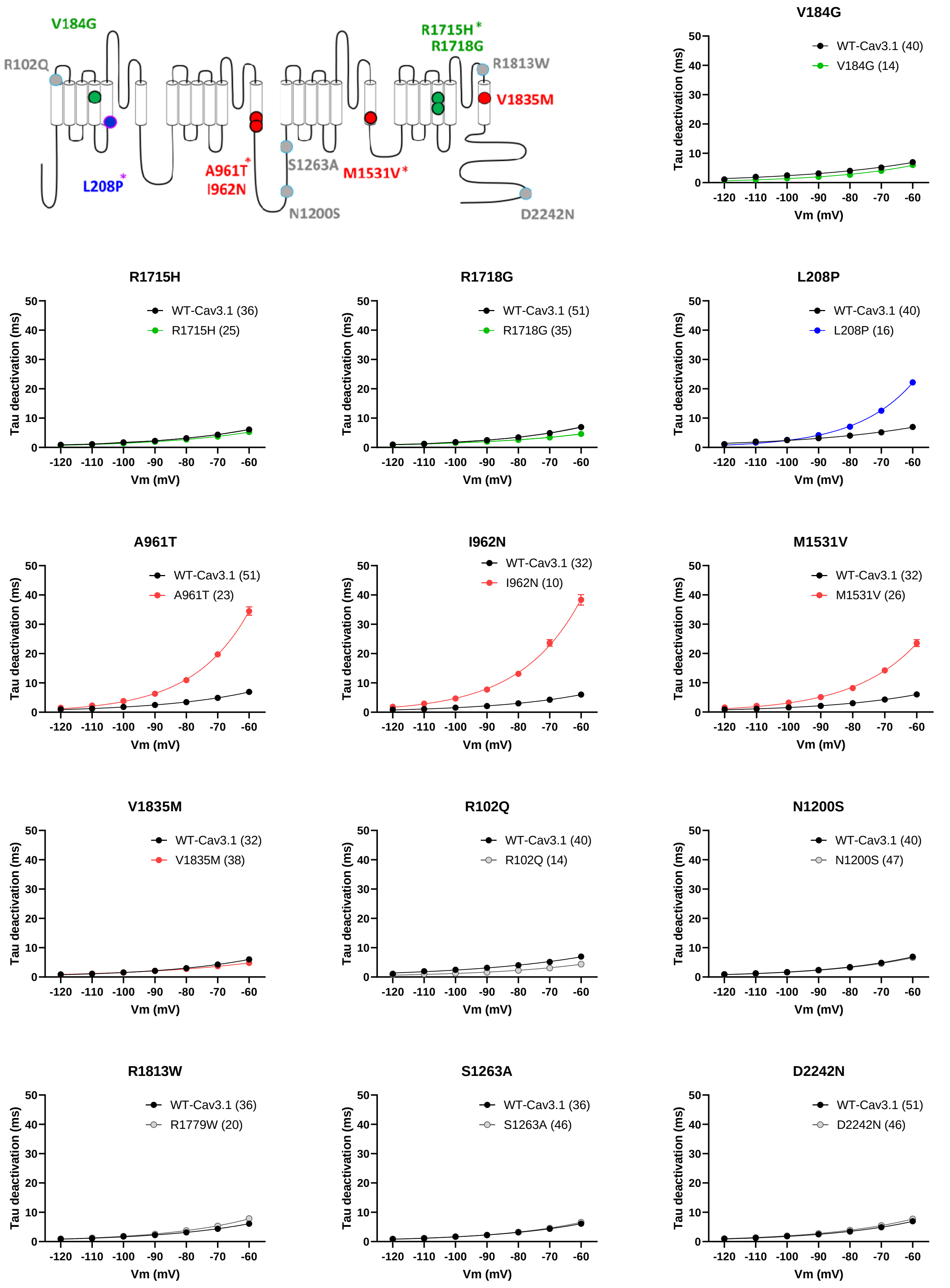

Supplementary Figure S3 : *deactivation kinetics in APC*

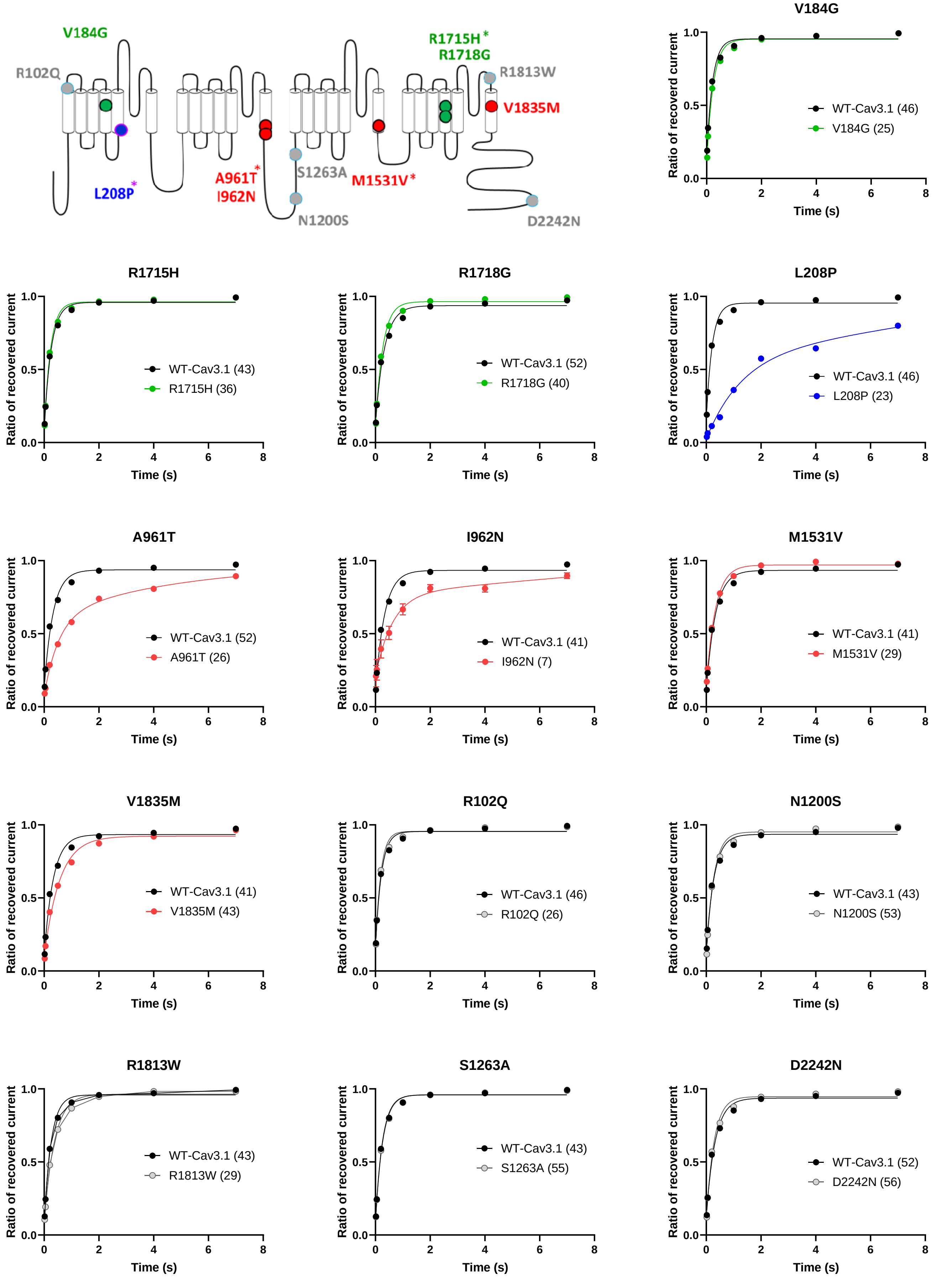

Supplementary Figure S4 : *recovery from inactivation in APC*

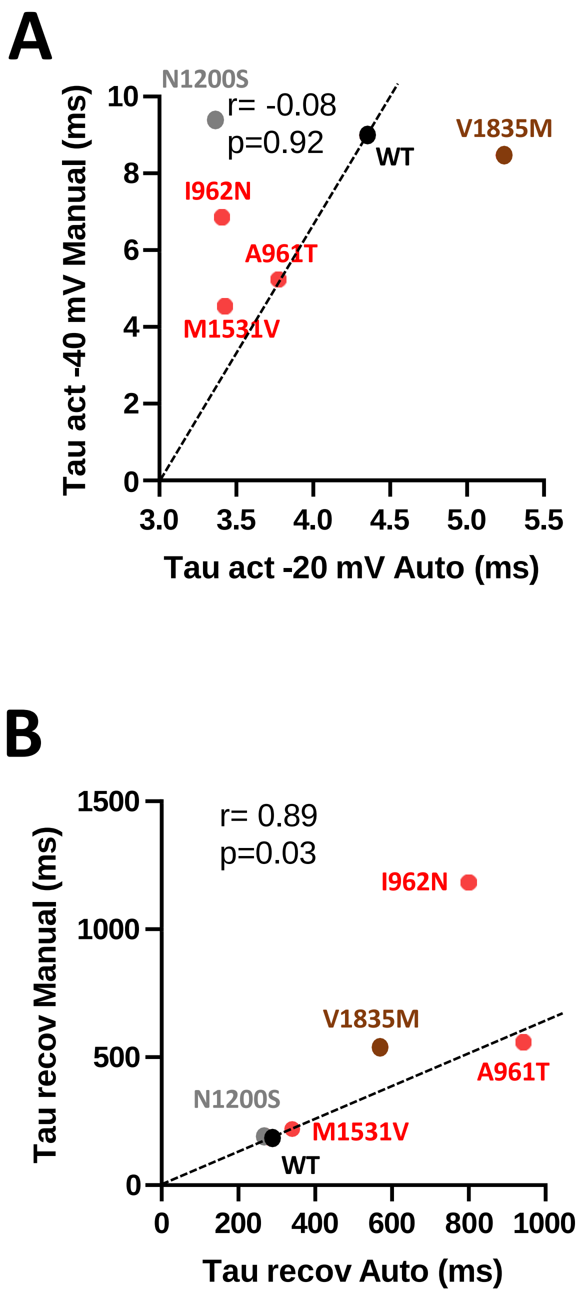

Supplementary Figure S5 : *APC vs MPC correlation for activation kinetics (***A***) and recovery from inactivation (***B***)*

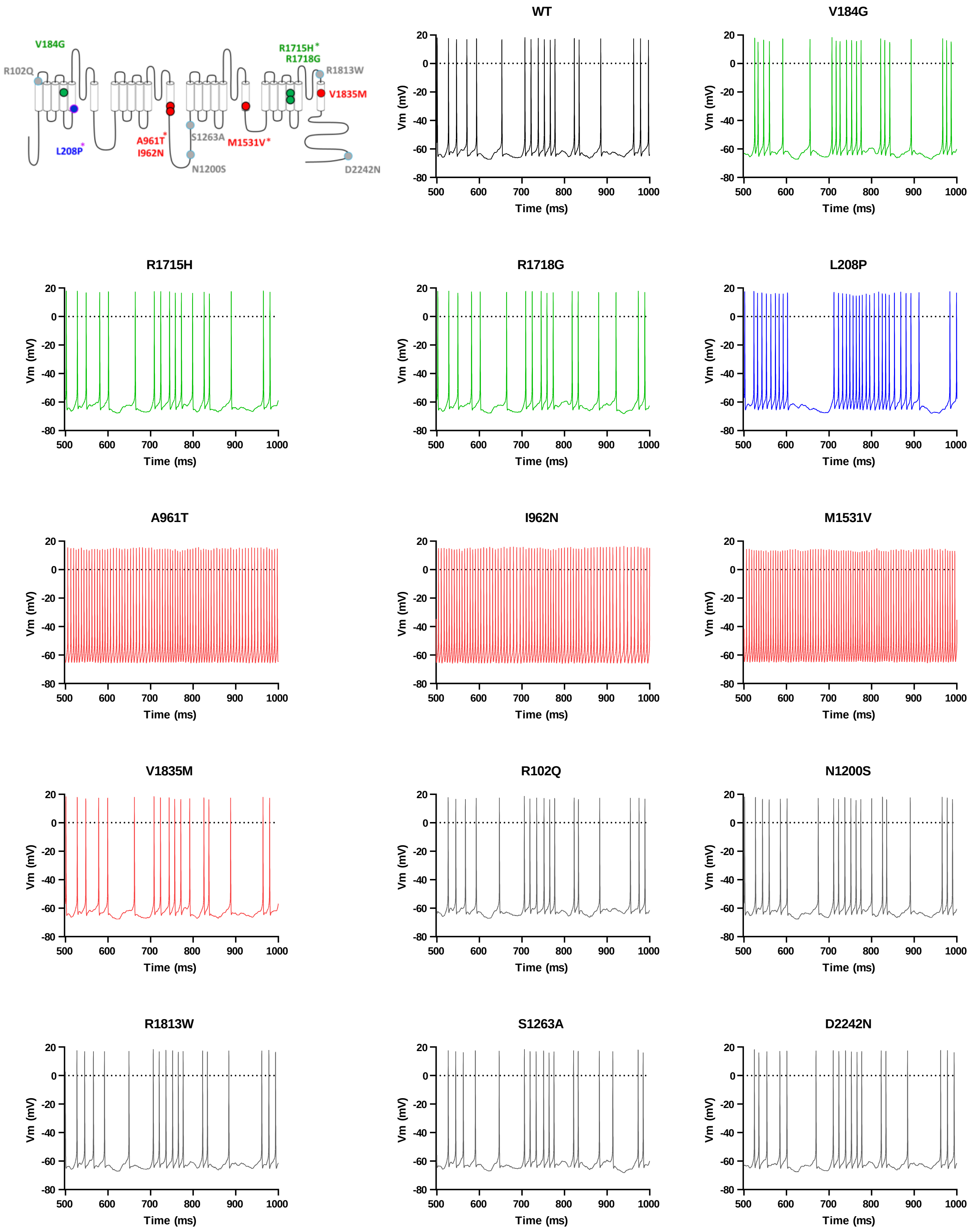

Supplementary Figure S6: *DCN modeling with APC electrophysiological parameters*

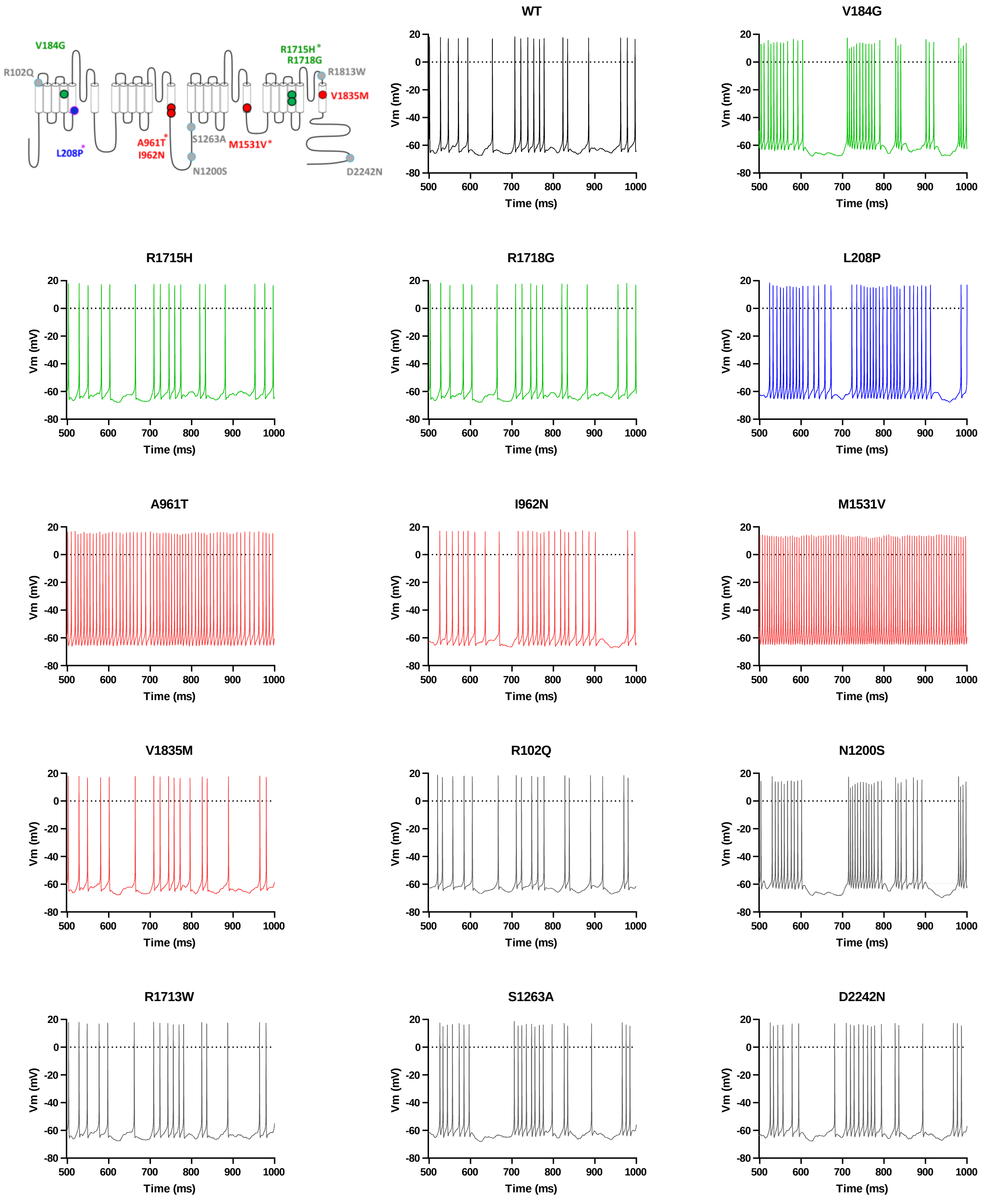

Supplementary Figure S7: *DCN modeling normalized for current density (APC param.)*

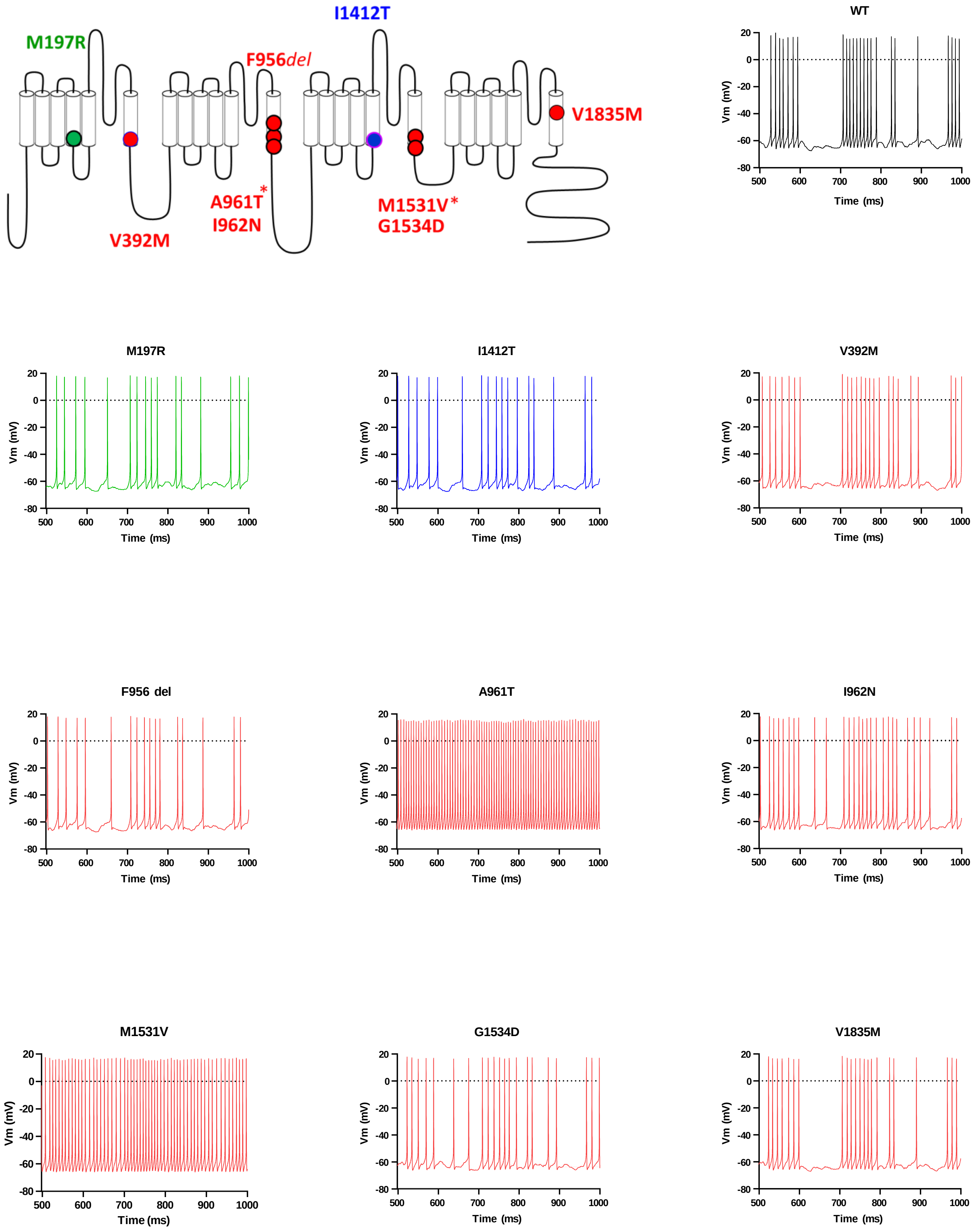

Supplementary Figure S8: *DCN modeling normalized for current density (MPC param.)*
